# Ankle-foot orthoses among children with cerebral palsy: a cross-sectional population-based register study of 8,928 children living in Northern Europe

**DOI:** 10.1186/s12891-023-06554-z

**Published:** 2023-06-02

**Authors:** Jessica Stockman, Guðbjörg Eggertsdóttir, Mark S. Gaston, Ira Jeglinsky-Kankainen, Sandra Julsen Hollung, Kirsten Nordbye-Nielsen, Philip von Rosen, Ann I. Alriksson-Schmidt

**Affiliations:** 1 Department of Clinical Sciences Lund, Orthopedics, Lund University, Remissgatan 4, Lund, 221 85 Sweden; 2 Centre for Clinical Research Sörmland, Uppsala University, Uppsala, Sweden; 3 SLF´S Rehabilitation Center- AEfingastöðin, Reykjavík, Iceland; 4 Cerebral Palsy Integrated Pathway, Royal Hospital for Sick Children, Edinburgh, Scotland United Kingdom; 5 Graduate School and Research, Arcada University of Applied Sciences, Helsinki, Finland; 6 Norwegian Quality and Surveillance Registry for Cerebral Palsy, Vestfold Hospital Trust, Tønsberg, Norway; 7 Department of Children’s Orthopedics, Danish Cerebral Palsy Follow-Up Program, Central Region Denmark, Danish Paediatric Orthopaedic Research Group, Aarhus University, Aarhus, Denmark; 8 Department of Neurobiology, Care Sciences and Society, Division of Physiotherapy, Karolinska Institutet, Alfred Nobels Allé 23, 141 83 Huddinge, Sweden

**Keywords:** Ankle-foot orthoses, Cerebral palsy, Children, Adolescents, CP-North

## Abstract

**Background:**

Cerebral palsy (CP) is an umbrella term where an injury to the immature brain affects muscle tone and motor control, posture, and at times, the ability to walk and stand. Orthoses can be used to improve or maintain function. Ankle-foot orthoses (AFOs) are the most frequently used orthoses in children with CP. However, how commonly AFOs are used by children and adolescents with CP is still unknown. The aims of this study were to investigate and describe the use of AFOs in children with CP in Sweden, Norway, Finland, Iceland, Scotland, and Denmark, and compare AFO use between countries and by gross motor function classification system (GMFCS) level, CP subtype, sex, and age.

**Method:**

Aggregated data on 8,928 participants in the national follow-up programs for CP for the respective countries were used. Finland does not have a national follow-up program for individuals with CP and therefore a study cohort was used instead. Use of AFOs were presented as percentages. Logistic regression models were used to compare the use of AFOs among countries adjusted for age, CP subtype, GMFCS level, and sex.

**Results:**

The proportion of AFO use was highest in Scotland (57%; CI 54–59%) and lowest in Denmark (35%; CI 33–38%). After adjusting for GMFCS level, children in Denmark, Finland, and Iceland had statistically significantly lower odds of using AFOs whereas children in Norway and Scotland reported statistically significantly higher usage than Sweden.

**Conclusion:**

In this study, the use of AFOs in children with CP in countries with relatively similar healthcare systems, differed between countries, age, GMFCS level, and CP subtype. This indicates a lack of consensus as to which individuals benefit from using AFOs. Our findings present an important baseline for the future research and development of practical guidelines in terms of who stands to benefit from using AFOs.

**Supplementary Information:**

The online version contains supplementary material available at 10.1186/s12891-023-06554-z.

Cerebral palsy (CP) is an umbrella term where an injury to the immature brain affects muscle tone and motor control, and oftentimes also posture and ability to walk and stand [1]. In several European countries, children with CP are given the opportunity to participate in a combined multidisciplinary follow-up program. The overall purpose of the program is to optimize function and prevent secondary complications throughout the lifespan through prevention rather than reactive care. A secondary goal is to evaluate specific interventions and treatments to provide evidence on the effectiveness to healthcare professionals working with individuals with CP [2, 3].

Orthoses, often in combination with other treatments, are used to improve or maintain function. Orthoses might enable activity and participation by facilitating movement, providing stabilization, and to lesser extent reducing pain in children with CP [4, 5]. In Sweden, Norway, Iceland, Finland, Scotland, and Denmark orthoses are prescribed free-of-charge by physiotherapists (PT), occupational therapists (OT), and physicians/orthopedic surgeons. The use of orthoses often varies based on the individual’s level of gross motor function and age [6, 7].

Ankle-foot orthoses (AFO) are the most frequently used orthoses in children with CP [6–8]. In Sweden, every other child with CP has been reported to use AFO [7]. AFOs provide direct control of the foot and ankle joints and indirectly affect the knee and hip joints [5, 6, 9]. The effects of a properly aligned orthosis on walking speed, energy cost of walking, stride, and step length are well substantiated [10–13]. However, there is still a lack of evidence of their effect on activities and participation [5, 13]. The decision to prescribe AFOs is made by the individual healthcare providers who are working with the children in collaboration with the children and their families. AFOs are introduced when tonus impairs function or risks causing misalignments, however, the age when most providers prescribe AFOs is unknown. Potential biomechanical and medical gains of using AFOs need to be weighed with the child/family’s goals and priorities [4, 5, 13, 14]. Thus far, the actual use of AFOs in a broader perspective has not been studied. Considering how common this treatment is around the world, the heterogeneity of individuals with CP, differences in opinions and traditions of healthcare providers combined with the lack of evidenced-based guidelines as to who would benefit from AFOs, and at what age, there is a need to learn more about which factors are associated with the use of AFOs. Descriptive population-based data on current practices in different countries can therefore provide important information in further guiding the development of this treatment. Having healthcare providers prescribe the treatment based on their own professional knowledge and beliefs may not harm the child per se. However, it involves great costs and resources, both for the family and the healthcare system.

In this study, we described the use of AFOs in children with CP in Sweden, Norway, Finland, Iceland, Scotland, and Denmark, and compared AFO use by country, gross motor function, CP subtype, sex, and birth year.

## Methods

### Participants and data collection

This study was based on cross-sectional register data from children ages 0–18 years included in the follow-up program and national register for individuals with CP in each participating country, Sweden (CPUP); Norway (NorCP); Iceland (CPEF); Scotland (CPIPS); and Denmark (CPOP) [15]. With the exception of Iceland, enrolment levels are high, ranging from 86 to 95% [16–19]. Generally, a diagnosis of CP is made at approximately 4 years of age. However, children with suspected CP are enrolled in the follow-up programs as early as possible in order to systematically receive proactive treatments and to manage symptoms. Hence, a small number of children are later found to have a different diagnosis than CP, or no diagnosis at all, and are then dropped from the programs. The Icelandic data were provided by two rehabilitation centres in the Reykjavik area and have an enrolment of approximately 39% based on estimates from the Icelandic CP population prevalence [20] at the time of the study. In Finland, all children with CP are followed regularly. However, Finland does not yet have a systematic, national surveillance program for children with CP [21]. Therefore, a cohort of children born 2000–2018, who had visited the University Hospital New Children´s Hospital in Helsinki and were assessed by a PT in the years 2017–2018 were included (n = 465). According to our estimates, this sample corresponds to about 18% of the Finnish population of children with CP at the time of the study [22, 23].

Due to the national regulations in some of the countries included, we were not permitted to share individual data across borders. Therefore, data from the latest PT assessments performed in 2017 or 2018 were de-identified and aggregated in each country and compiled per birth year, by CP subtype, sex, gross motor function, and the use of AFOs for all children born between 2000 and 2018 in the register/cohort. Based on the assessment schedules, the participants are assessed once or twice per year or every other year (depending on age and level of gross motor function) by PTs and OTs [24] and data are recorded in country specific databases. This research collaboration between the Nordic countries falls under the research program CPNorth – Living Life with Cerebral Palsy in the Nordic Countries (https://www.arcada.fi/en/research/key-research-activities/cp-north) [15].

### Variables

AFO included all types of orthoses that start below the knees, end on the feet, and provide direct control over the foot and ankle joints. Use of AFOs was dichotomized (yes/no). Birth years were categorized into three groups: 2000–2005, 2006–2011, and 2012–2018. Gross motor function was classified according to the Gross Motor Function Classification System (GMFCS) levels (I-V or not classified). GMFCS level I indicates the highest gross motor function and level V the lowest [24]. CP subtype was classified according to the Surveillance of Cerebral Palsy in Europe’s guidelines [25] and recorded as spastic (unilateral or bilateral), ataxic, dyskinetic, and not classified.

### Statistical analyses

The use of AFOs was presented as percentages (%) and the raw numbers of the denominators (n) were presented by country, birth year group, GMFCS level, CP subtype, and sex. Linearity of the participants’ AFO use by birth year was first inspected through a bar chart of merged data from all countries birth year data. Because there was a peak in AFO use for children born 2006–2011, the decision was made to categorize the participants into three birth year groups. Logistic regression was used to compare the use of orthoses (yes/no) among countries adjusted for birth year group, GMFCS level, CP subtype, and sex. In the regression analysis, data were included only for those children who had subtypes and GMFCS level recorded. Sweden, with the most participants, was set as the reference country.

As the data were aggregated, it was not possible to adjust for sex, CP subtype, and GMFCS level in the same model. Instead, one model that included only country and birth year group, which were available for all participants was used. Subsequently, we adjusted for CP subtype, GMFCS level, and sex in three logistic regressions with AFO use as the dependent variable with 95% confidence intervals (CI) and level of statistical significance set to *p <* 0.05. Given the small sample from Iceland (N = 70), logistic regressions were performed with and without the Icelandic data included. However, the results were similar, and therefore the findings were presented with the Icelandic data included. SPSS v27 was used for all analyses.

## Results

The proportion of children reported to use AFOs was highest in Scotland with 57% of 1,955 children and lowest in Denmark with 35% of 1,196 (Table 1). The proportion of children using AFOs in the three birth year groups was lowest among the children born 2000–2005 in all countries, except for in Denmark, where the children born 2012–2018 were the least likely to have AFOs.

**Table 1:** Proportions reporting Ankle Foot Orthoses by country, birth year group, cerebral palsy subtype, level of gross motor function classification system (GMFCS), and sex with the denominator (n)

Country	Sweden	Norway	Finland	Iceland	Scotland	Denmark
**Total** **Confidence Interval 95%**	51% (3,851) 49–53%	56% (1,387) 53–59%	43% (465) 39–48%	39% (74) 28–50%	57% (1,955) 54–59%	35% (1,196) 33–38%
**Birth year**						
2012–2018 2006–2011 2000–2005	52% (987) 56% (1,559) 45% (1,305)	54% (391) 61% (699) 47% (297)	60% (167) 34% (170) 34% (128)	33% (15) 47% (34) 32% (25)	60% (440) 60% (853) 49% (662)	29% (529) 40% (573) 44% (94)
**Subtype**						
Ataxic Dyskinetic Spastic bilateral Spastic unilateral Not classified	20% (127) 69% (340) 61% (1,088) 39% (888) 49% (1,408)	23% (47) 67% (104) 66% (598) 49% (612) 23% (26)	0% (3) 37% (51) 51% (132) 42% (219) 42% (60)	0% (1) 67% (6) 45% (44) 20% (15) 25% (8)	24% (34) 51% (194) 64% (839) 52% (627) 55% (261)	12% (26) 29% (59) 48% (506) 26% (574) 23% (31)
**GMFCS level**						
GMFCS I GMFCS II GMFCS III GMFCS IV GMFCS V Not classified	33% (1,704) 47% (588) 68% (362) 75% (564) 72% (633) 0	42% (715) 66% (238) 73% (98) 78% (134) 74% (192) 20% (10)	35% (204) 57% (99) 60% (45) 48% (56) 35% (57) 0% (4)	22% (27) 26% (19) 80% (10) 50% (14) 75% (4) 0	42% (730) 62% (387) 74% (185) 72% (258) 62% (395) 0	20% (603) 43% (198) 58% (85) 54% (136) 57% (170) 0% (4)
**Sex**						
Boys Girls	50% (2,229) 52% (1,622)	55% (803) 57% (584)	44% (262) 43% (203)	45% (42) 31%(32)	56% (1,113) 58% (834)	34% (700) 37% (496)
Unknown	0	0	0	0	13% (8)	0

**Fig. 1:**
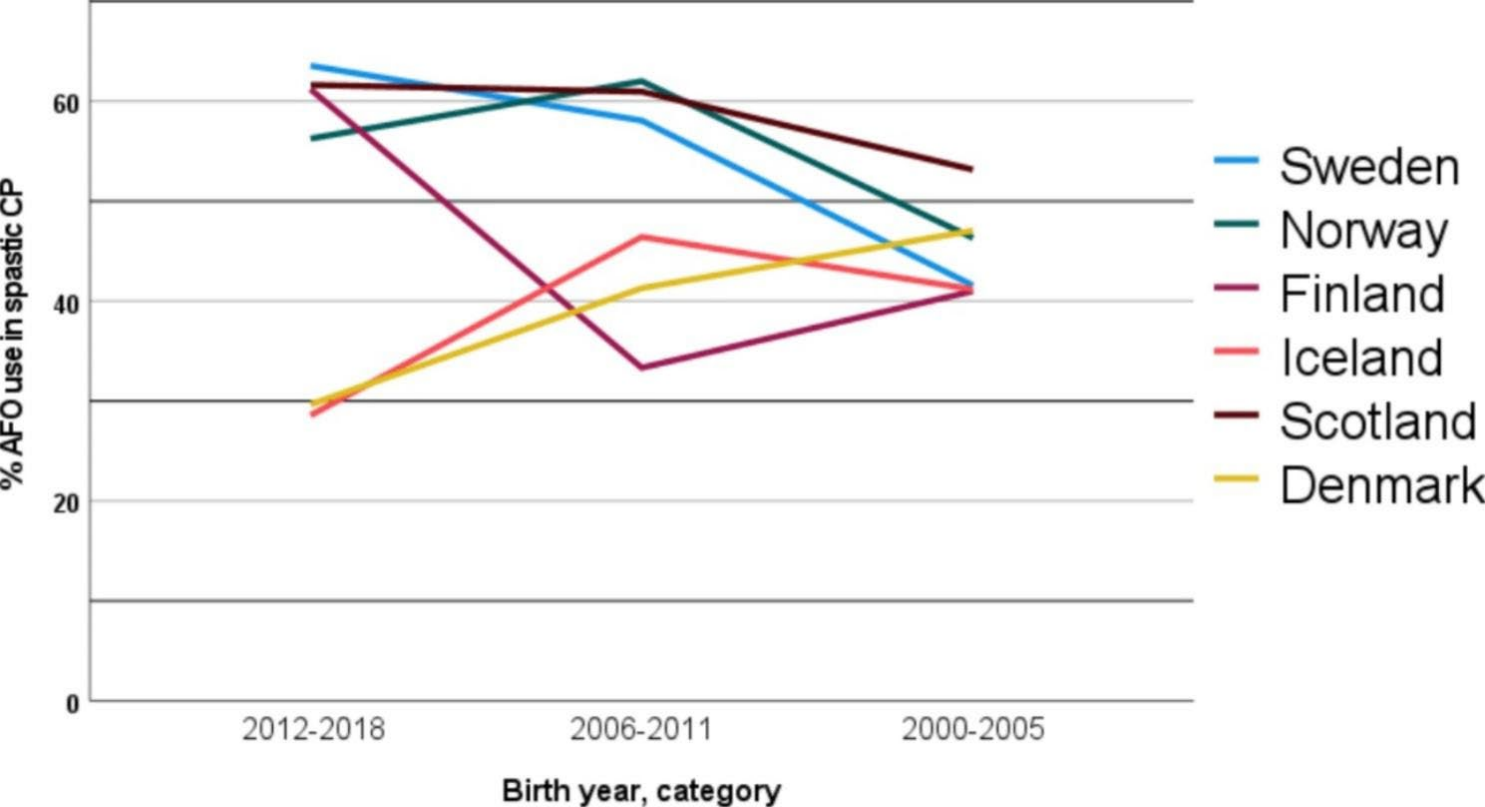
Proportion between age and country for ankle-foot orthoses (AFO) use in children with spastic cerebral palsy (CP) presented by three birth year groups

Children with dyskinetic CP were most likely to use AFOs in Sweden, Norway, and Iceland. This stands in contrast to Finland, Scotland, and Denmark, where children with bilateral spastic CP most often used AFOs. When combining unilateral and bilateral CP into one “spastic CP” category, birth year group and country-related differences emerged more clearly. Denmark and Iceland reported in 2017-2018 the lowest proportions of AFOs in the youngest children, whereas in Sweden, Norway, and Scotland the lowest proportion of AFO use was in the oldest children (Fig. 1). All data aggregated by country and birth year group can be found in Appendix 1.

When categorized by GMFCS level, the proportion of children using AFOs decreased to 20–42% in GMFCS level I compared to 58–80% in level III (Table 1).

Reported AFO use did not statistically significantly differ between boys and girls; more girls had AFOs in all countries except for in Iceland and Finland. In Iceland, 45% (19/42) of the boys and 31% (10/32) of the girls were reported to use AFOs. In Finland, 44% (115/262) of the boys used AFOs compared to 43% (87/203) of the girls.

The use of AFOs differed statistically significantly by country (Table 2). The difference was smaller after adjusting for CP subtype but greater when adjusting for GMFCS levels (Table 2). Including GMFCS level in the model (Model 3, Table 2), Norway (odds ratio (OR) 1.37 CI: 1.20–1.56) and Scotland (OR 1.20 CI: 1.07–1.35), had statistically significantly higher odds of AFO use than Sweden adjusted for country and birth year, which was not the case in the model where we adjusted for CP subtype only (Model 2, Table 2).

**Table 2:** The use of ankle foot-orthoses reported by country, birth year group (Model 1), subtype of cerebral palsy (Model 2), gross motor function classification system (GMFCS) level (Model 3), and sex (Model 4) with the reference group in italics

	Model 1		Model 2		Model 3		Model 4	
	Odds Ratio (95% Confidence Interval)	*p*	Odds Ratio (95% Confidence Interval)	*p*	Odds Ratio (95% Confidence Interval)	*p*	Odds Ratio (95% Confidence Interval)	*p*
**Country**								
*Sweden*								
Norway	1.17 (1.03–1.32)	**0.014**	1.11 (0.97–1.28)	**0.141**	1.37 (1.20–1.56)	**< 0.001**	1.17 (1.03–1.32)	**0.014**
Finland	0.72 (0.60–0.88)	**0.001**	0.68 (0.55–0.85)	**0.001**	0.74 (0.60–0.91)	**0.004**	0.72 (0.59–0.88)	**0.001**
Iceland	0.65 (0.41–1.04)	0.071	0.52 (0.31–0.86)	**0.011**	0.58 (0.35–0.95)	**0.032**	0.65 (0.41–1.04)	0.071
Scotland	1.26 (1.13–1.41)	**< 0.001**	1.09 (0.96–1.25)	0.176	1.20 (1.07–1.35)	**0.002**	1.27 (1.13–1.42)	**< 0.001**
Denmark	0.48 (0.42–0.56)	**< 0.001**	0.43 (0.37–0.50)	**< 0.001**	0.50 (0.43–0.57)	**< 0.001**	0.48 (0.42–0.55)	**< 0.001**
**Birth year**								
*2000–2005*								
2006–2011	1.53 (1.38 − 1.69)	**< 0.001**	1.68 (1.50–1.89)	**< 0.001**	1.70 (1.53–1.90)	**< 0.001**	1.54 (1.38–1.70)	**< 0.001**
2012–2018	1.32 (1.18–1.48)	**< 0.001**	1.61 (1.40–1.85)	**< 0.001**	1.42 (1.26–1.60)	**< 0.001**	1.33 (1.18–1.49)	**< 0.001**
**CP Subtype**								
*Spastic unilateral*								
Ataxic			0.34 (0.25–0.47)	**< 0.001**				
Dyskinetic			2.01 (1.70–2.41)	**< 0.001**				
Spastic Bilateral			2.17 (1.95–2.41)	**< 0.001**				
**GMFCS level**								
*I*								
II					2.22 (1.97–2.51)	**< 0.001**		
III					4.43 (3.74–5.23)	**< 0.001**		
IV					4.84 (3.74–5.60)	**< 0.001**		
V					3.84 (3.38–4.37)	**< 0.001**		
**Sex**								
*Male*								
Female							1.09 (1.00–1.18)	0.054
**N**	8928		7134		8910		8920	
**Constant**	-0.204	< 0.001	-0.579	< 0.001	0.976	< 0.001	-0.243	< 0.001
**Nagelkerke**	0.035		0.102		0.158		0.036	

Denmark and Finland had statistically significantly lower AFO use ORs in all four models (Denmark range of OR 0.43–0.50 and Finland range of OR 0.68–0.74) than Sweden. In Iceland, the ORs were lower for AFO use than Sweden but this was only statistically significant when adjusting for CP subtype (OR 0.52 CI 0.31–0.86) and GMFCS level (OR 0.58 CI 0.35–0.95). Differences in AFO use between the sexes were close to statistically significant (p = 0,054) with higher odds of using AFOs for the girls (OR 1.09 CI 1.00-1.18).

## Discussion

In this study, we investigated the prevalence of AFO use in Sweden, Norway, Iceland, Finland, Scotland, and Denmark by birth year group, CP subtype, GMFS level, and sex. Despite having similar healthcare systems and follow-up programs for children with CP, the reported use of AFOs differed substantially among the countries, even after controlling for birth year, CP subtype, GMFCS level, and sex. The results may reflect the heterogeneity of the study population and the subjective decision of when and to whom AFOs should be prescribed. The findings could also be explained by country-specific preferences and traditions in terms of who is believed to benefit from using AFOs. To explain the reasons for the observed differences, more research is needed. The use of AFOs in Sweden has been studied previously by Wingstrand et al. in 2014 [7] and our results on the Swedish prevalence of AFO use was identical to that report (51%), which indicates that the proportion of AFO use has been stable over the last 7 years. Moreover, Wingstrand et al. reported that the goals set for AFO use as maintenance or improvement of range of motion were reached in 70% (73% when the goals were set to specifically improve function) [7], which means that one third to one quarter of all children with AFOs in Sweden might not reach their AFO treatment goals.

Prescribing orthoses to children with CP when they may not actually be beneficial to the child is probably not harmful or detrimental to their musculoskeletal care. However, it might restrict the child in terms of participation and carrying out certain physical activities. For example, a child with AFOs might have better stability in standing but may not be able to put on his/her shoes without assistance, which might infringe on their independence. In Firouzeh et al’s review in 2019 they describe the lack of evaluating AFOs in participation and activity outcomes which is important given that more than every second child with CP use AFOs in some countries. Anti et al. suggested already in 2006 that daily routine and floor mobility in small children might be challenged by AFO use [13, 26]. This should be kept in mind when interpreting the results from this study where the greatest proportion of AFO use is among the youngest children (0–6 year of age at the time of data collection) in Finland and Scotland. Furthermore, the children born 2000–2005 in this study have the lowest proportion of AFO use in all included countries except for Denmark (Appendix 1). This despite the fact that it most likely would be the older children who might benefit the most from AFOs in terms of reduced energy consumption, increased step length, and walking speed as older children in general are more likely to participate in leisure and school activities outside the home. It is, of course, possible that the children born 2000–2005 might have worn AFOs at a younger age and decided to stop for some reason, a question worth further investigation.

Owen (2020) discusses the importance of goalsetting in terms of activity and participation in addition to biomechanical control [5], and how it is important to monitor all of these factors over time. Healthcare systems are struggling in many countries in terms of financial resources and staff and need to ensure that the limited healthcare resources are used appropriately and provide the most cost-effective evidence-based treatment. However, not prescribing orthoses to children who would benefit from them could have significant negative implications for the child’s musculoskeletal health, both in the short and long term. Nevertheless, the findings from the current study seem to indicate that the current use of AFOs in six countries is inconsistent and variable.

The GMFCS level was the strongest predictor in terms of estimating AFO use in children and adolescents with CP and data on GMFCS level were recorded for almost every child in the study (> 99,9%). The GMFCS level associated with the greatest proportion of AFO use, differed among the countries, however. For instance, in Sweden and Norway, individuals at GMFCS level IV were more likely to report AFO use, compared to individuals at GMFCS level III in Finland, Iceland, Scotland, and Denmark. The fact that there were no statistically significant differences between the proportions of AFO use by sex is encouraging given that there are no apparent reasons as to why the use of AFOs should differ between boys and girls.

The predictive value determined by Nagelkerke R was low overall (< 16), indicating that CP subtype and GMFCS level are not strong factors in terms of predicting which children with CP will use AFOs, although both are important factors in the prescribing process. This highlights the need for clearer evidence-based guidelines to help providers and families decide who should be prescribed AFOs. If the work on when to use lower extremity orthoses begun by Owen [5] is developed and implemented as general recommendations, the results of this study will provide an opportunity to evaluate changes in AFO use based on new guidelines.

### Limitations

The fact that we had to use aggregated data affects the ability to adjust for country, gross motor function, CP subtype, sex, and birth year group in the same model, which might have given us a more precise view of who is the most typical AFO user. Although not included as an aim, it would still have been helpful to have had more information available regarding if and when a child might have used AFOs in the past. Despite this study being population-based in Scotland, Norway, Denmark, and Sweden, the small, perhaps not representative, number of participants in Iceland and Finland might limit the generalizability. In addition to this, a small number of individuals from Iceland were included, which means that the data from a few individuals had a large impact on the group percentages. However, this also reflects the small overall population of Iceland. Furthermore, given that the CP subtype is not always determined before the child’s fourth birthday, the subtypes for the children under five years of age should be interpreted with caution. In addition, Sweden had a large percentage of missing diagnoses of CP subtype (38%), which has been an ongoing problem due to lack of neuropediatricians. This is currently being remedied and future studies including Swedish CPUP data should have less missing data on CP subtype. Finally, some children with suspected but not diagnosed CP might have been included in the study given that also children younger than 4–5 years of age, the age when a diagnosis of CP is generally made, were included. However, we know from many years’ experience that most young children included in the follow-up program do eventually get a diagnosis of CP.

## Conclusion

This study contributes to our understanding of the use of AFOs in children with CP in Northern Europe, where AFOs are provided free of charge at the point of use. We found that the AFO use in children with CP in countries with similar healthcare systems, differed between countries, age, level of gross motor function, and CP subtype. Our findings present a baseline for the future research development of practical guidelines in terms of who stands to benefit from using AFOs.

## Electronic supplementary material

Below is the link to the electronic supplementary material.

Supplementary Material 1

## Data Availability

All data generated or analyzed during this study are included in this published article [and its supplementary information files.

## List of Abbreviations

AFO: Ankle-foot orthoses
CP: Cerebral palsy
GMFCS: Gross motor function classification system
OT: Occupational therapist
PT: Physiotherapist
