# Supplementary material for: Ankle-foot orthoses among children with cerebral palsy: a cross-sectional population-based register study of 8,928 children living in Northern Europe
Source: BMC Musculoskelet Disord. 2023 Jun 2;24:443. doi: 10.1186/s12891-023-06554-z (PMC10236765; doi:10.1186/s12891-023-06554-z)
Supplement: Supplementary file 1 — Supplementary Material 1 [file 12891_2023_6554_MOESM1_ESM.docx]

**Appendix 1.** Proportions and frequencies of children with ankle-foot orthoses, shown as value for the denominator (n), by age group, country, cerebral palsy subtype, gross motor function classification system (GMFCS) level, and sex.

|  | **Birth year 2012-2018** | | | | | | **Birth year 2006-2011** | | | | | |
| --- | --- | --- | --- | --- | --- | --- | --- | --- | --- | --- | --- | --- |
| Country | **Sweden** | **Norway** | **Finland** | **Iceland** | **Scotland** | **Denmark** | **Sweden** | **Norway** | **Finland** | **Iceland** | **Scotland** | **Denmark** |
| % Total | 52% (987) | 54% (391) | 60% (167) | 33 % (15) | 60 % (440) | 29% (529) | 56% (1559) | 61 % (699) | 34% (170) | 47 % (34) | 60 % (853) | 40% (573) |
| Ataxic | 12% (17) | 22% (9) | 0% (1) | 0 | 75% (4) | 20% (10) | 19% (42) | 28% (29) | 0% (1) | 0 | 20% (20) | 7% (14) |
| Dyskinetic | 71% (28) | 63% (24) | 69% (13) | 100% (1) | 54% (41) | 30% (20) | 68% (148) | 73% (52) | 37% (19) | 100% (2) | 62% (77) | 30% (33) |
| Spastic bilateral | 71% (126) | 69% (158) | 77% (30) | 38% (8) | 69% (172) | 36% (203) | 68% (468) | 67% (300) | 42% (55) | 48% (21) | 65% (375) | 57% (250) |
| Spastic unilateral | 55% (99) | 45% (185) | 57% (86) | 17% (6) | 53% (159) | 25% (269) | 47% (393) | 57% (308) | 28% (80) | 29% (7) | 56% (286) | 27% (273) |
| Not classified | 48% (717) | 13% (15) | 54% (37) | (0/0) | 56% (64) | 22% (27) | 51% (508) | 30% (10) | 33% (15) | 50% (4) | 64% (95) | 33% (3) |
| GMFCS I | 40% (438) | 47% (196) | 52% (73) | 27% (11) | 47% (169) | 19% (265) | 38% (699) | 47% (370) | 25% (80) | 30% (10) | 46% (332) | 20% (304) |
| GMFCS II | 48% (139) | 52% (62) | 81% (32) | 33% (3) | 71% (86) | 44% (85) | 52% (264) | 71% (126) | 47% (38) | 17% (6) | 61% (174) | 42% (93) |
| GMFCS III | 68% (106) | 63% (38) | 72% (18) | 100% (1) | 71% (41) | 46% (46) | 76% (134) | 87% (38) | 44% (9) | 80% (5) | 78% (77) | 78% (32) |
| GMFCS IV | 66% (134) | 69% (42) | 65% (23) | 0 | 66% (56) | 31% (61) | 81% (214) | 86% (73) | 37% (19) | 50% (10) | 80% (114) | 73% (60) |
| GMFCS V | 59% (170) | 77% (43) | 47% (19) | 0 | 67% (88) | 40% (68) | 77% (248) | 74% (92) | 36% (22) | 100% (3) | 68% (156) | 69% (84) |
| Not classified | 0 | 20% (10) | 0% (2) | 0 | 0 | 0% (4) | 0 | 0 | 0% (2) | 0 | 0 | 0 |
| Boys | 55% (420) | 48% (250) | 58% (88) | 25% (8) | 59% (244) | 28% (315) | 57% (630) | 63% (389) | 40% (108) | 53% (19) | 61% (506) | 39% (326) |
| Girls | 49% (567) | 65% (141) | 63% (79) | 43% (7) | 63% (192) | 31% (214) | 54% (929) | 59% (310) | 23% (62) | 40% (15) | 60% (343) | 41% (247) |
| Unknown |  |  |  |  | 0% (4) |  |  |  |  |  | 25% (4) |  |

|  | **Birth year 2000-2005** | | | | | |
| --- | --- | --- | --- | --- | --- | --- |
| Country | Sweden | Norway | Finland | Iceland | Scotland | Denmark |
| % Total | 45% (1305) | 47 % (297) | 34% (128) | 32 % (25) | 49 % (662) | 44% (94) |
| Ataxic | 24% (68) | 11% (9) | 0% (1) | 0% (1) | 0% (10) | 0% (2) |
| Dyskinetic | 70% (164) | 61% (28) | 16% (19) | 33% (3) | 37% (76) | 17% (6) |
| Spastic bilateral | 53% (494) | 59% (140) | 45% (47) | 47% (15) | 59% (292) | 53%(53) |
| Spastic unilateral | 27% (396) | 32% (119) | 38% (53) | 0% (2) | 44% (182) | 38% (32) |
| Not classified | 49% (183) | 100% (1) | 0% (8) | 0% (4) | 46% (102) | 0% (1) |
| GMFCS I | 22% (567) | 24% (149) | 27% (51) | 0% (6) | 31% (229) | 21% (34) |
| GMFCS II | 38% (185) | 70% (50) | 41% (29) | 30% (10) | 57% (127) | 45% (20) |
| GMFCS III | 59% (122) | 68% (22) | 56% (18) | 75% (4) | 72% (67) | 43% (7) |
| GMFCS IV | 74% (216) | 63% (19) | 36% (14) | 50% (4) | 65% (88) | 67% (15) |
| GMFCS V | 76% (215) | 72% (57) | 19% (16) | 0% (1) | 52% (151) | 67% (18) |
| Not classified | 0 | 0 | 0 | 0 |  | 0 |
| Boys | 45% (572) | 48% (164) | 32% (66) | 47% (15) | 47% (363) | 42% (59) |
| Girls | 45% (733) | 45% (133) | 37% (62) | 31%(32) | 52% (299) | 46% (35) |
| Unknown |  |  |  |  |  |  |
